# Neurogenesis in the trunk and brain of the milkweed bug *Oncopeltus fasciatus*: insights beyond holometabolan models

**DOI:** 10.1186/s12983-025-00593-z

**Published:** 2025-12-10

**Authors:** Nitzan Alon, Ariel D. Chipman

**Affiliations:** The Department of Ecology, Evolution and Behavior, The Alexander Silberman Institute of Life Sciences, The Hebrew University of Jerusalem, 9190401 Jerusalem, Israel

**Keywords:** Neurogenesis, Insects, Pre-gnathal segments, Evolution, Central nervous system, Brain, Segmentation

## Abstract

Current research on insect neurogenesis is focused on Holometabola, which undergo a derived developmental mode that includes metamorphosis, leading to a partial representation of the range of neurogenesis patterns in the embryonic insect nervous system. In this paper, we investigate neurogenesis in the hemimetabolous insect *Oncopeltus fasciatus*, focusing on both the trunk and brain regions. Our findings reveal early neuroblast formation in the brain, followed by a second phase in the whole embryo. Notably, we did not find gene expression patterns consistent with pro-neural clusters in *Oncopeltus*, and the brain exhibits characteristics that do not exist in the trunk. We also highlight similarities between segmentation and neurogenesis, proposing that spatial cues play a key role in this differentiation, as both the head and the thorax segment simultaneously. Our analysis suggests that brain development follows a separate trajectory from trunk neurogenesis in at least one neuroblast population that develops earlier than their counterparts. These findings diverge from the commonly presented view of insect neurogenesis and offer new insights into the ancestral roles of key genes involved in neural development.

**Supplementary Information:**

The online version contains supplementary material available at 10.1186/s12983-025-00593-z.

## Background

Insect neurogenesis has been a focus of study for decades [1–4], providing the basis for understanding many neuronal processes, and gene functions [5–7]. However, studies on this process have long focused on holometabolan insects, leading to two major gaps in our knowledge.

First, because most of these studies were performed on Holometabola [1–4, 8, 9], which have a derived biphasic life history that includes metamorphosis [8, 9], our picture of insect neurogenesis is of a derived process. This is a significant point since there are substantial differences in nervous system structure between the holometabolous larva and adult [3, 9, 10], and most of the neurogenesis process occurs in two waves: once during embryogenesis and a second time during the pupal stage [4, 11, 12]. Focusing on embryonic neurogenesis potentially misses many important differentiation events that only occur during metamorphosis. Examples of species that display the ancestral direct developing life history are essential for drawing more general conclusions about the development and evolution of insect nervous systems.

Second, many studies have focused on the trunk region [3, 5, 9, 13, 14]. The neurogenesis processes within the embryonic insect brain have been described in detail mostly on holometabolan models [5, 9, 15], with a few exceptions [8, 9, 16]. Knowledge of the development of the insect brain is still fragmentary compared to the ventral nerve cord, which has been studied in greater detail. The brain has unique neurogenic characteristics and unique structures, and is also the part of the nervous system that changes the most during metamorphosis [3, 9, 10], further increasing the knowledge gap.

For these reasons, we have chosen to focus on neurogenesis in both the trunk and the brain of the hemimetabolous insect *Oncopeltus fasciatus*, comparing and contrasting neurogenesis between these two regions. We also highlight some interesting variations from the *Drosophila* textbook example of insect neural development.

### The arthropod central nervous system and brain development

The arthropod brain is a three-part structure that is a synapomorphy for Arthropoda [17, 18]. It is located in the three most anterior segments of the head, known as the pre-gnathal segments (PGS) or procephalon. The brain is followed by the segmented ventral nerve cord with paired ganglia along the trunk (in insects, this includes the gnathal segments of the head). These ganglia sometimes shift along the body axis and fuse to join other ganglia [3, 9].

The formation of the three-part brain includes morphogenetic movements and structural transformation of neuropils during organogenesis [17, 19]. This transformation can be rather extreme, consisting of folding of the early neural structures along different axes [5, 9] and resulting in a dorsal or even posterior-dorsal location of the anteriormost region, the protocerebrum, relative to the main body axis [3, 9]. This is known as the “bend and zipper” model [19].

The ectodermal domain that gives rise to the trunk neural precursors is specified around the time of gastrulation and has well-documented molecular characteristics and structural markers. This is a medio-ventral region in the trunk, which will give rise to the ventral nerve cord and ganglia in insects, as well as in all other arthropods that have been studied [5, 13, 20, 21].

**Fig. 1:**
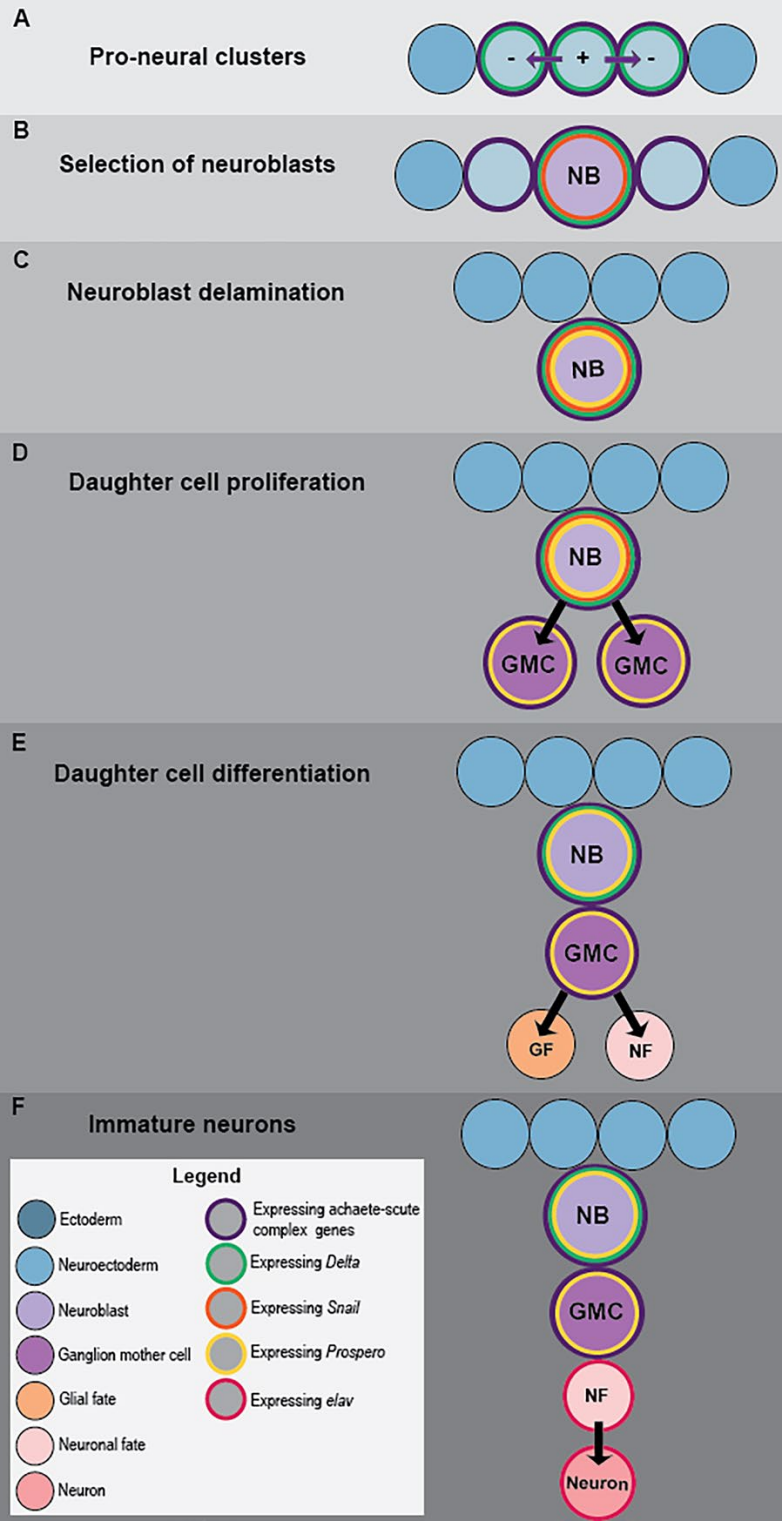
Summary of neural precursor specification in the ventral nerve cord of insects: Schematic drawing showing the early stages of neurogenesis in insects, including cell identity changes and gene expression patterns. **A** Pro-neural clusters are established within the neuroectodermal domain (blue circles), neurogenic potential is maintained by bHLH genes (purple outline), and lateral inhibition mediated by Delta-Notch (green outline) occurs within the cluster. Direction of inhibition is indicated by a plus sign in the inhibiting cell and arrows towards the inhibited cells marked by a by minus sign. **B** Single neuroblasts (light purple circle) are selected out of the cluster and enlarge. Delta-Notch signaling upregulates bHLH genes in the neuroblast, and inhibits the expression of both in the neighboring cells. The neuroblasts begin to express *Snail* (orange outline), as it is required for asymmetrical cell division. **C** The neuroblasts delaminate from the ectoderm into the embryo and begin to express *prospero* (yellow outline). **D** The neuroblasts divide asymmetrically to create the smaller ganglion mother cells (GMCs, dark purple circles), *Snail* facilitates the asymmetrical transfer of *prospero* RNA to these daughter cells, which activates terminal differentiation. **E** The daughter cells (GMCs) undergo one additional symmetrical mitosis, producing two sibling cells. These cells can adopt either a glial fate (orange circles) or a neuronal fate (pink circles). *Prospero* in the GMCs promotes cell cycle exit and triggers differentiation. **F** newborn neurons begin to differentiate and specialize (dark pink circles), as well as express pan-neural genes such as *elav* (Magenta outline). Abbreviations: GF—glial fate, GMC, ganglion mother cell, NB—neuroblast, NF—neural fate

During insect neurogenesis, BMP/Wnt and Delta-Notch signaling determine the pattern and number of proneural clusters. A proneural cluster is a group of neuroectodermal cells where proneural genes are initially expressed and neurogenic potential is maintained by a well-studied set of transcription factors of the basic helix-loop-helix (bHLH) family, in addition to other genes [4, 6, 7, 22–24] (Fig. 1). Within these clusters, lateral inhibition mediated by Delta-Notch signaling takes place. This leads to the selection and specification of typically one single cell within each cluster to become a neuroblast. Each of these neuroblasts is identifiable by the expression of a combination of marker genes [1, 2, 13, 25, 26] (Fig. 1B). These specify the identity of the neuroblast’s daughter cells depending on the time of the mother cell’s creation and its location. This selected neuroblast then delaminates (Fig. 1A–B) [5, 8, 9] from the neuroectoderm and enters a phase of proliferation. Insect neuroblasts are neural stem cells that divide asymmetrically to produce ganglion mother cells (GMCs) as their progeny (Fig. 1D–F). Insect neuroblasts typically enlarge before they sink into the embryo and are significantly larger than neighboring cells (Fig. 1B–E) [5, 8].

During the proliferation phase, a series of transcription factors, notably snail (Fig. 1B–D), control migration and asymmetrical division into a basal and an apical cell (Fig. 1C–D). Snail is required for the asymmetric cell divisions of the neuroblasts as it is needed for the spindle rotation that precedes the production of GMCs [5]. Snail activity influences the asymmetrical distribution of the cell-fate determinant genes [27, 28], such as *prospero* (*pros*) [5]. The apical daughter cell remains active as a neuroblast, while the basal cell divides to generate neurons and glial cells [27]. The expression of *pros* terminates mitosis and trigger neural differentiation [5, 29]. The progeny can be either neurons or glial cells. Newborn neurons (Fig. 1F), while not yet identifiable morphologically, already express pan-neuronal genes such as *elav* (Fig. 1F) and a unique combination of transcription factors identifying their lineage and determining their identity [1–3, 5].

The neurons arising within the PGS display a key difference compared to those of the trunk [15, 30, 31]. In the insect brain, there are two types of neuroblasts, known as type I and type II, whereas in the trunk there are only type I neuroblasts. Type I neuroblasts divide and generate a series of ganglion mother cells expressing *pros* [29]*.* The ganglion mother cells then divide to give rise to neurons and/or glia [5, 14]. Type II neuroblasts generate immature intermediate progenitor cells that proliferate before producing ganglion mother cells, adding another stage that enables them to have larger progenies [8, 16, 31, 32]. Type II neuroblasts are brain-specific, and were found to contribute mainly to the brain's central complex [8, 16, 31]. This highlights the importance of looking at neurogenesis in the brain and not just in the trunk.

### *Oncopeltus fasciatus* as a model

The large milkweed bug, *Oncopeltus fasciatus* belongs to Hemiptera, the “true bugs”, a hemimetabolous insect order, which is the closest sister group to Holometabola. This places it in an ideal position as an outgroup for the study of events of the early evolution of Holometabola [33]. The *O. fasciatus* hatchling nervous system is a miniature of the adult one, and no new structures are added during the insect’s life [34], which means complete organogenesis of the nervous system occurs during the embryonic stage. These attributes make it a good model for understanding the ancestral state of the insect brain and nervous system developmental processes.

No copy of the gene *asense* has been found in Hemiptera*.* In the two hemipteran species *Cimex lectularius* and *Halyomorpha halys* there is only a single gene from the *achaete-scute* family, named *ash* (*acheate-scute homolog*) [35, 36] (Supplementary Fig. 1). This matches our finding in the *O. fasciatus* genome, where we found only a single copy of *ash* in the published genome (NCBI accession number: PRJNA229125), and no copy of *asense.* Therefore, we used this gene as a neurogenesis marker, as it is the most likely to take over the role of *asense.*

Here, we show that the neurogenesis process in *O. fasciatus* differs from what has been found in previous studies on holometabolous insects in terms of gene expression and dynamics and discuss how this may affect our viewpoint on nervous system development and evolution in insects. Our results also support the idea that certain modes of neurogenesis are unique to the PGS outside of Holometabola and show that the relative speed of neurogenesis in the PGS is faster than in the trunk*.* These results re-contextualize previous data and encourage us to reexamine our assumptions about insect neurogenesis.

## Methods

### Animal culture and egg collection

*Oncopeltus fasciatus* individuals were kept in plexiglass cages at a temperature of 25 °C and a 14/10-h light/dark cycle. Each cage contained a few dozen individuals. We collected eggs by placing cotton balls in cages with sexually mature individuals for a predetermined time frame. We then kept the collected eggs in a 25 °C incubator until the eggs reached the desired stage of development.

### Digoxigenin-labeled probes

We designed the primers for all relevant genes from the published *O. fasciatus* genome. (NCBI accession number: PRJNA229125), [37]. An additional, unpublished new assembly was used for verification, as the analysis of this assembly is still in progress. The primer design was done using the Geneious software package (Biomatters). We synthesized the probes using Sigma-Aldrich T7 RNA kit and Digoxigenin-labeled ribonucleotides. For *Delta*, we used previously published primers for generating RNA probes [38]. Gene identifiers from the *O. fasciatus* genome [37] and primers are listed below:

*Snail* (OFAS025184): forward AGCCCAAATCCAAGGAGACT, reverse TCGCAGTACTTGCAGGAGAA

*Ash* (OFAS005280): forward CGCTTACAACGGTATGCAGC, reverse AGGCCGTAGAACAGGGAGAT

*Pros* (OFAS013318): forward CCTTCTTCCTGCCCTGAAGG, reverse ATCAGAGGAGGAAGCCGACT

*Elav* (OFAS009793): forward CTACACTTGCCATCCCACCT, reverse ACTGGGAGAACAGCACCAGT

### Embryo preparation

We submerged *O. fasciatus* eggs of the desired age in tap water and boiled them for 3 min, followed by placing on ice for 5–7 min. We replaced the water with Heptane: formaldehyde (12%) in phosphate-buffered saline with 0.1% Tween-20 (PBT). The embryos were then mildly shaken for 20 min. Embryos were stepped into methanol with increasing concentration.

For embryos meant for HCR-FISH, we performed the process described above, but we replaced 12% formaldehyde in PBT with 4% paraformaldehyde (PFA) in phosphate-buffered saline (with no tween) after replacing the water with heptane, as described above. The embryos were manually dissected from the yolk immediately following this. All embryos were stored in methanol until the in-situ hybridization or antibody staining.

### Chromogenic in situ hybridization

Fixed embryos were transferred to PBT solution in a stepwise manner and manually dechorionated in PBT. They were then transferred to hybridization buffer (Formamide, 20XSSC- pH = 4.5, SDS 20%, blocking reagent- Roche, Yeast t-RNA- Invitrogen, Heparin) for over an hour at 60 °C. Embryos were then hybridized with 330–1000 ng probe in 1 ml hybridization buffer at 60 °C overnight. The embryos were then washed a few times in hybridization buffer and then with PBT. Blocking was carried out in a mixture of 10% Normal Goat Serum in PBT, overnight at 4 °C. After blocking, embryos were transferred into AP (Alkaline Phosphatase) buffer and AP-conjugated antibodies against Digoxigenin (Sigma-Aldrich, 11093274910) were added to them at a concentration of 1:2000 and left overnight at 4 °C. The embryos were then stained by transferring them to a staining buffer (BM-purple, Sigma-Aldrich 11442074001) and were allowed to develop between three hours and overnight. Embryos were then transferred stepwise to 70% glycerol in PBT for storage at 4 °C, dissection and visualization. Stained germband stage embryos were manually dissected from the yolk in 70% glycerol and mounted on microscope slides.

### HCR-FISH

All reagents are from the Molecular Instruments HCR-FISH kit (Hybridization Chain Reaction—Fluorescent in Situ Hybridization). The protocol is based on the Patel Lab protocol [39]. Fixed embryos were transferred to PBT solution in a stepwise manner, and permeabilized in 500 µl detergent solution at 37 °C for 30 min. The detergent was removed, and the embryos were transferred to pre-warmed hybridization buffer/ 100 µg/ml salmon sperm DNA (Invitrogen 15,632,011), at 42 °C for 1 h. The probe solution was prepared with 0.8–1.5 µl probe from stock, in 200 µl hybridization buffer, warmed to 37 °C. In the case of double staining, multiple probes with different hairpin types were used at once.

The hybridization buffer was replaced with the probe solution and placed to incubate overnight at 37 °C. The probe solution was then removed, and the embryos were washed in pre-warmed wash buffer at 37 °C twice for 16 min, then washed in SCCT 5% 4 times at room temp, for 5 min. The embryos were submerged in an amplification buffer at 37 °C for 30 min. The hairpin mix was made from heat-shocked 1.8 µl hairpin solution at 95 °C for 90 s, followed by cooling in the dark back to room temp and mixing all relevant hairpins in 200 µl amplification buffer. The pre-amplification solution was removed, and embryos were left in the dark overnight at 37 °C. The hairpin mix was then removed, and the embryos were washed with SCCT 5% 5 times for 15 min and then mounted in glycerol on a microscope slide.

### Antibody staining

Fixed embryos were blocked with 10% NGS (Normal Goat Serum) + 5% DMSO (dimethyl sulfoxide) in PBT. After adding the primary Anti-alpha tubulin (1:50; DSHB, 12G10, Uniprot ID: P41351), or anti-phosphorylated histone 3 (1:50; Sigma-Aldrich, NCBI accession no. NP003484.1) antibodies, the embryos were incubated for 1 h at room temp, then overnight at 4 °C. Then, they were incubated at room temp for an additional hour before 5 washes in PBT, 15 min each. The embryos were then incubated for 30 min in 10% NGS in PBT at room temp, before adding the secondary antibody (1:200, Alexa-fluor 448, anti-mouse, Invitrogen A-11029). They were then incubated in the dark for an additional 2–3 h. Finally, they were washed in PBT, 4 times for 10 min each, with DAPI (4′,6′-diamidino-2′-phenylindole) added to the second wash. Embryos were stepwise transferred to 75% glycerol in PBS, dissected and mounted on slides. In our samples the anti- alpha-tubulin staining marks what appears to be in most cases cell outlines, which are clearly larger and have a different shape than the nuclei stained by DAPI.

### Microscopy and imaging

Images of slide-mounted embryos were taken using a Nikon DS-Fi3 V12 mounted on a Nikon Eclipse 80i. White balance and color balance were done using NIS Elements imaging software. Stitching, alignments and background removal were done using Adobe Photoshop.

HCR images were taken using an Olympus FV1200 confocal based on an IX-83 inverted microscope (Olympus, Japan), using a 10×/0.4 or 40×/0.95 air objective. Confocal fluorescence images of DAPI and Alexa 488 anti-alpha-tubulin and DAPI and Alexa 488 anti-phosphorylated histone 3were acquired using the same setup. For higher magnifications, the brain lobe on the left or right side of was picked based on image quality, and mirror-flipped to fit the figure orientation if needed. Images were processed using Fiji [40]. We selected the Z-stacks containing the signal for each fluorescent channel, as determined subjectively by eye. For virtual slices, we selected the Z-stacks starting with the apical layer of the embryo and ending with the basal layer, as determined subjectively by eye. Each slice contains two sequential scans.

After projecting stacks, we adjusted the contrast and brightness of each channel to maximize the signal or reduce noise. Due to differences in intensity in different parts and layers of the embryo, in some images the DAPI overshines the antibody staining and in some regions the opposite occurs.

Cell diameters of putative neuroblasts and a control group were measured based on the anti-alpha tubulin staining using the measure function in Fiji. Finally, we false-colored the channels and merged them into a single RGB file.

## Results

### Overview of germband-stage morphogenesis

**Fig. 2:**
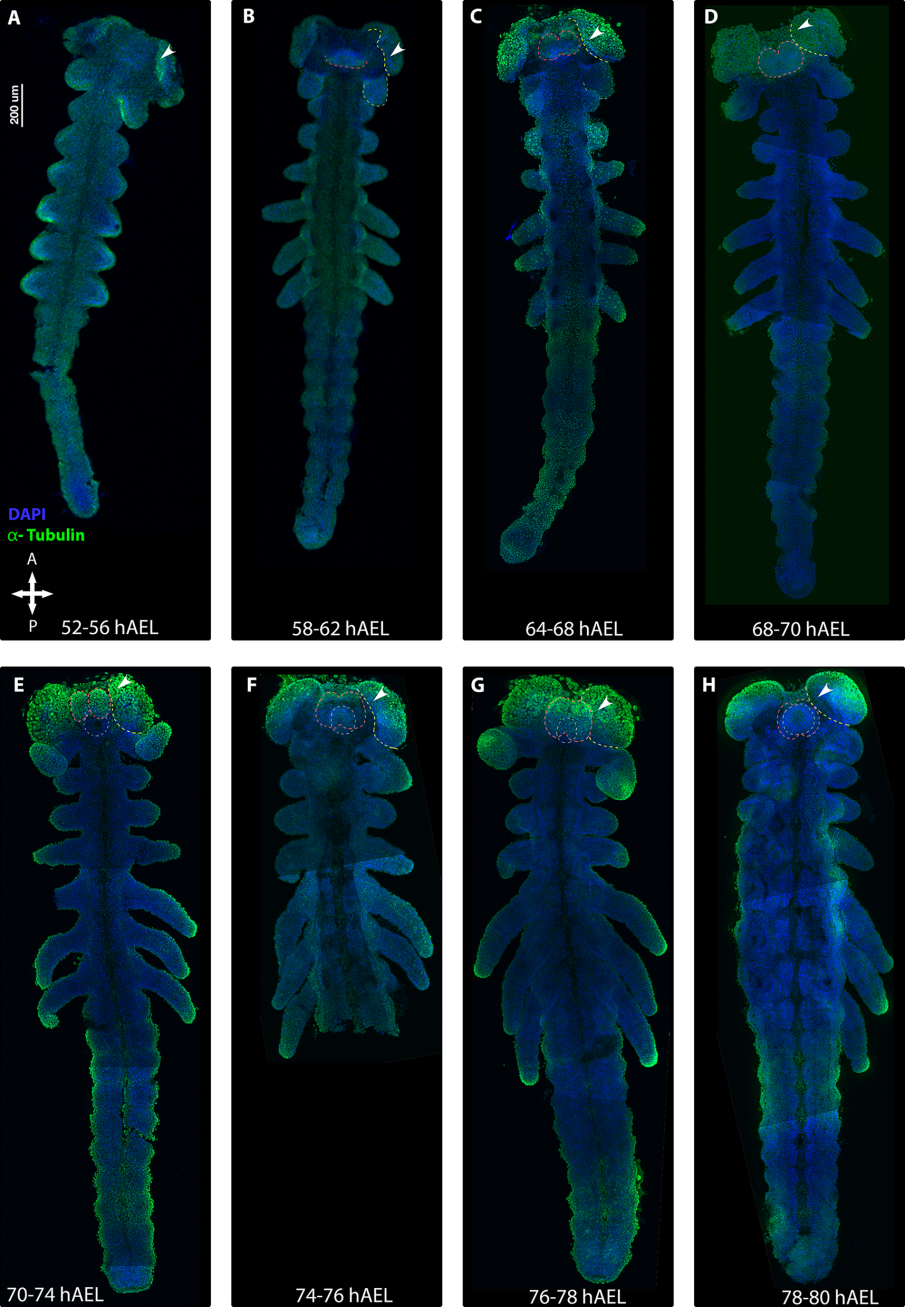
A time series of α-tubulin antibody stains during the mid to late germband stage in *Oncopeltus fasciatus*. Anterior towards the top. α-tubulin (green) and DAPI (blue). Dashed lines mark morphological structures. **A** 52–56 hAEL, head lobes begin to fold, creating a head crease (white arrowhead). The thorax shows a uniform, flat cell layer. **B** 58–62 hAEL, head fold deepens (white arrowhead), and the brain lobes start to bubble (yellow dashed lines) and the labrum starts forming around the midline of the PGS (dashed red line). Thoracic limbs begin to telescope out from the thoracic segments but the midline still appears uniform. **C** 54–68 hAEL, the head fold thickens, and the labrum is fully formed (dashed white lines). Thoracic limbs continue to telescope, and the midline region gets thinner. **D** 68–70 hAEL, the head lobes are fully formed (dashed yellow lines) and limbs have finished elongating. **E** 70–74 hAEL Segmentation in the abdomen is complete, and the mouth (dashed white line) is visible underneath the labrum (dashed red outline). In the brain lobes (dashed yellow line), the internal structure- most likely the future ocular lobes -is now clear even in full projection. **F** 74–76 hAEL segmental cell blocks form along the trunk, and the head lobes begin to bend ventrally, bringing the mouth (dashed white line) further beneath the labrum. The midline is thinned to a single-cell layer. **G** 76–78 hAEL Germband retraction begins as the abdomen thickens. **H** 78–80 hAEL. The segmental cell blocks are now formed along the whole trunk. In the brain lobes, the internal structure is clear, and the mouth is now fully beneath the labrum. The separate fluorescent channels can be found in Supplementary Fig. 2A and B

**Fig. 3:**
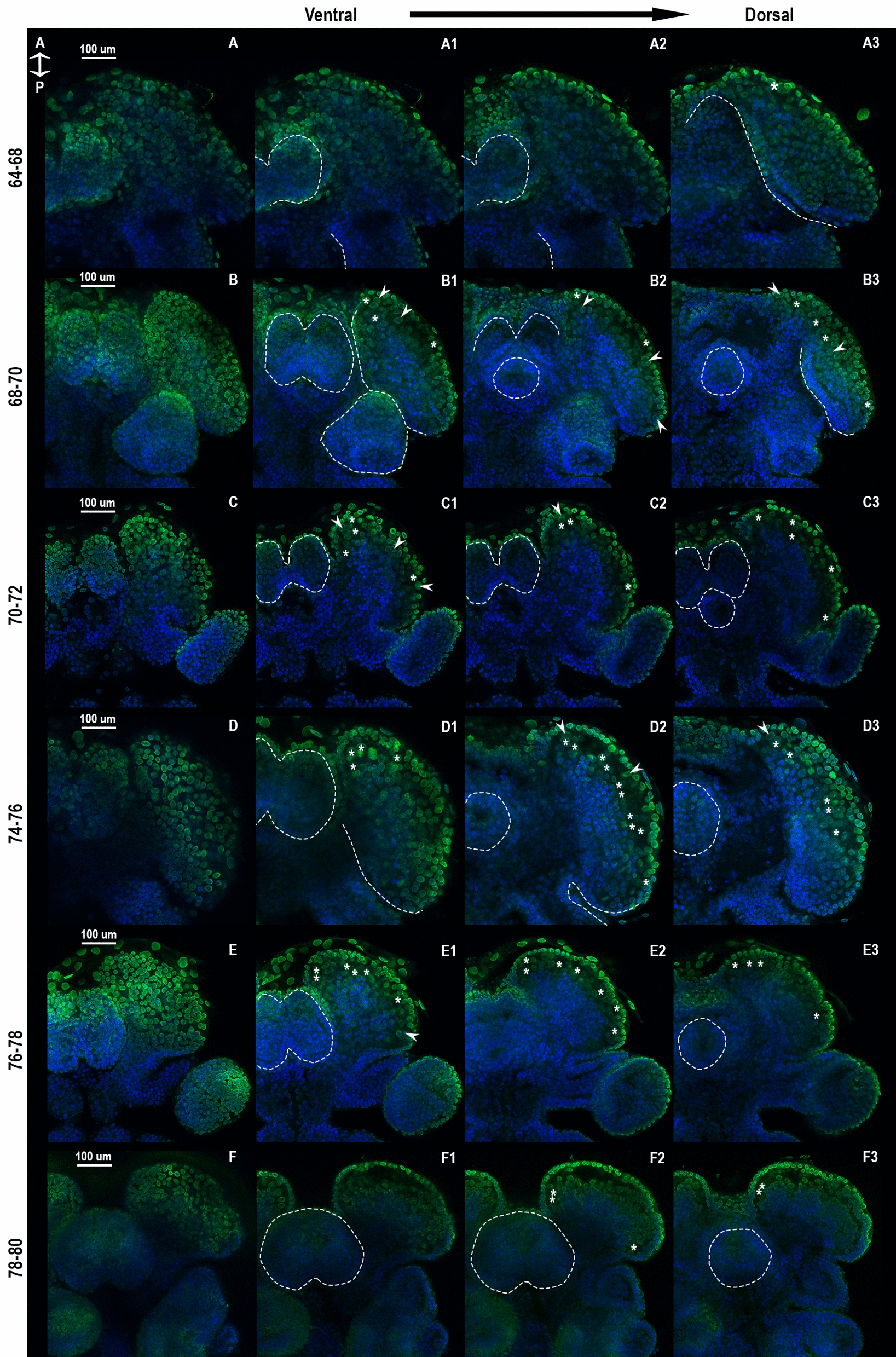
Series of α-tubulin antibody-stained digital sections of the PGS of *Oncopeltus fasciatus*. The anterior-right head region of germband stage embryos, stained for α-tubulin (green) and DAPI (blue). In each row, the left panel is a full projection of a confocal stack, and columns 1–3 are increasingly deep optical slices. Viewed ventrally, anterior towards the top. Morphological structures outlined by dashed lines, morphologically distinct neuroblasts (> 8 µm in diameter) marked by asterisks, cells with visibly dividing nucleus (DAPI) marked by arrowheads. **A** 1–3: The head fold thickens, and the labrum is fully formed (dashed lines). **B** 1–3: The head brain lobes are fully formed, in addition to the labrum. The mouth starts to move anteriorly (dashed white lines), and a growing population of neuroblasts is visible (asterisks), but still mostly appears in the outermost layers of the brain lobe. **C** 1–3: The head fold is no longer visible due to the thickness of the brain lobes. More neuroblasts appear in the inner layers or delaminate from the ectoderm (the outermost layer). A few can be seen dividing parallel to the surface (white arrowheads). **D** 1–3: The brain lobes begin to bend ventrally, bringing the mouth (white dashed circle, D2–D3) further beneath the labrum. The internal structure continues to grow as can be seen by the concentration of nuclei stained by DAPI. Most distinct neuroblasts are at the lateral parts of this structure. **E** 1–3: The mouth is now fully beneath the labrum, all visible distinct neuroblasts are now inside the brain lobe. **F** 1–3: In the brain lobes, the internal structure is now fully formed, and most distinct neuroblasts are no longer visible. Additional slices and single channels—in Supplementary Fig. 3A–C

At early germband formation and during the beginning of abdominal segmentation (48–56 h after egg laying, or hAEL), the embryo is composed of uniform-looking layers of cells, and neuroblasts are not distinguishable based on size (cytoskeleton staining used for approximation) or position (Fig. 2A). The sole markers of future cell fates are gene expression patterns (see next section- Fig. 3). However, the brain lobe has already started to fold over itself (Fig. 2A, arrowhead). Limbs and antennal lobes begin to telescope out at around 58–68 hAEL (Fig. 2B), and the labrum starts to form. By 64–70 hAEL, the brain lobes have grown in volume over the previous fold (Fig. 2C–D). As the segmentation in the abdomen ends (Fig. 2E), limbs are significantly extended, as are the antennal lobes, and the mouth is visible. The brain lobes and labrum are inflated and have an internal structure underneath the outer cell layer. As germband retraction begins (Fig. 2F–H), cellular structures appear along the midline, forming a thin region in the midline with thicker margins, in which morphological segmental cell blocks begin to be seen. Head morphogenesis begins as well, as the mouth moves into place underneath the labrum, and the brain and antennal lobes start to bend.

**Fig. 4:**
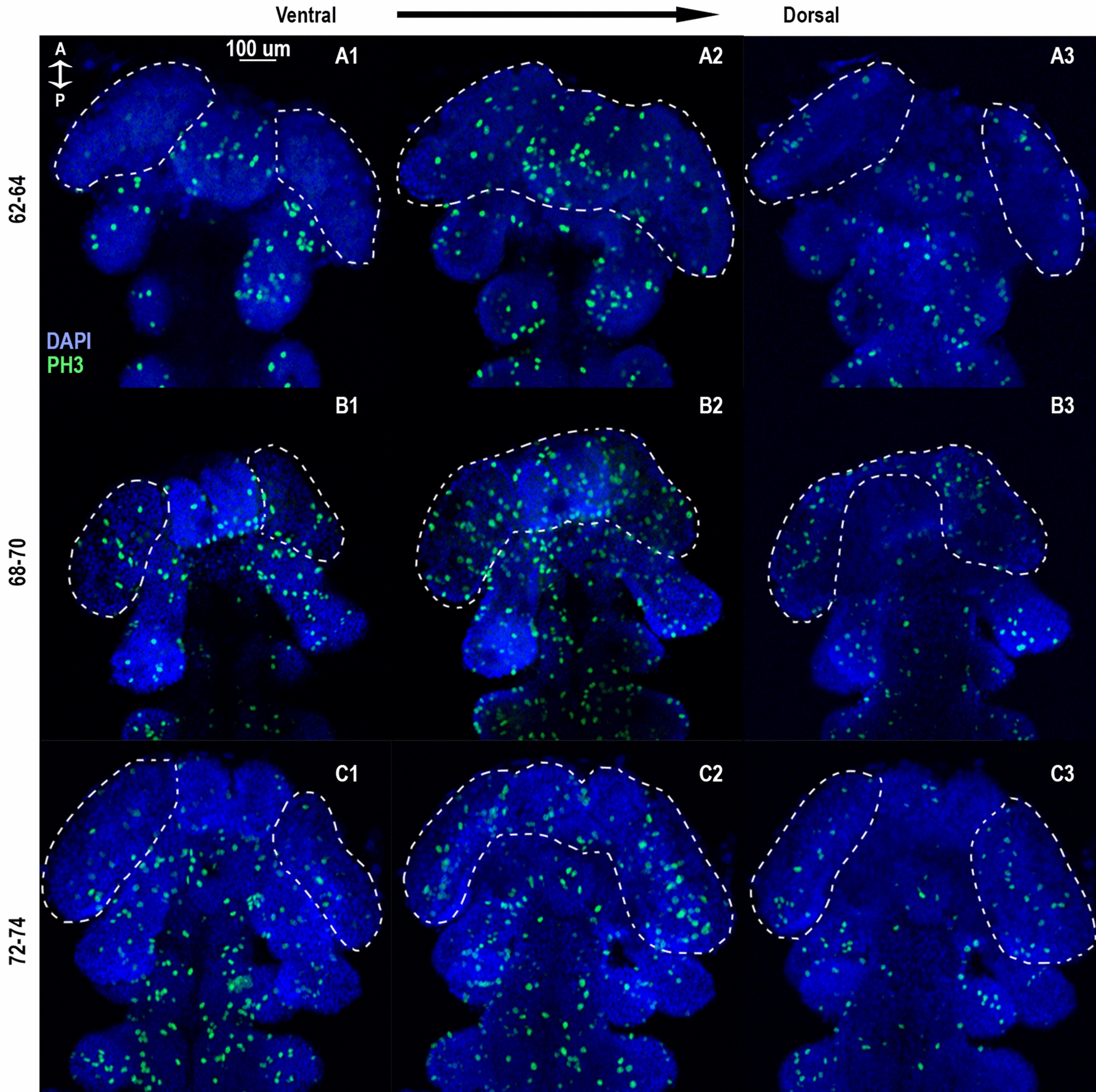
Series of Ph3 antibody-stained digital sections of the PGS of *Oncopeltus fasciatus.* The anterior head region of germband stage embryos. Dividing cells, marked by phosphorylated histone 3 are in green. Nuclei, stained with DAPI are in blue. Columns 1–3 show increasingly deeper optical sections. Anterior towards the top. Dashed lines mark the outline of the brain regions. **A** 1–3: Most dividing cells can be seen in the innermost section of the head, at the boundaries of the headfold, and at the tips of the antennal lobes, as well as around the mouth. **B** 1–3: While some cells can be seen dividing in the outer layers of the embryo (the ectoderm), most cell divisions still occur in the innermost layers, and some divisions seem to take place around the mouth and inside the forming structure in the head, as well as around the head fold. **C** 1–3: Divisions can be seen mostly in the inner structure inside the brain lobes, compared to the outer layers of the embryo's head. While in the gnathal segments, most of the divisions are visible in the top cell layer. Single channels – in Supplementary Fig. 4A and B

The first evidence for morphologically distinct putative neuroblasts (cells which have a diameter of over 8 µm based on the outline of their cytoskeleton compared to the average 4.23 µm in the rest of the embryo head, as measured in our samples, chosen at random. N = 50, stdv = 1.11) appears in the brain lobes around 64–68 hAEL (Fig. 3, asterisks). These can be found at the most anterior part of the brain lobes, in the inner layers of the PGS, underneath the ectoderm, while the labrum is forming in the ventral-most part of the head, noted here in order not to be confused with the inner brain structure, as it may obscure it. We determined their identity as neuroblasts based on neural markers in the same area (see below). As evidenced by the digital sections in Fig. 3, this gives the *Oncopeltus* head a multilayered structure. The anterior of the embryonic A-P axis begins to bend dorsally (Fig. 3A–B). While there is an increase in cell division in the whole brain region compared to the rest of the head (Fig. 4, Supplementary Fig. 4C), the population of morphologically distinct neuroblasts increases in number by 68–72 hAEL and some can be seen in deeper layers of the brain lobes (Fig. 3B–C), and even dividing (Fig. 3B–C, white arrowheads). By 74–78 hAEL (Fig. 3D–E), most distinct neuroblasts are in the inner layers of the brain lobes, and there is a large number of nuclei that show a dense structure developing inside the brain lobes. The structure of the head lobes continues folding, bringing cells located anteriorly to a more medial position. As shown by gene expression data in the next section, we suggest these are the GMCs and newborn neurons, beginning to form the insect brain. This structure grows and thickens as neurogenesis continues from the neuroectoderm until about 80 hAEL (Fig. 3F), at which point most putative neuroblasts are no longer visible. The mouth has moved to its position underneath the labrum.

### Neural differentiation in the trunk

**Fig. 5:**
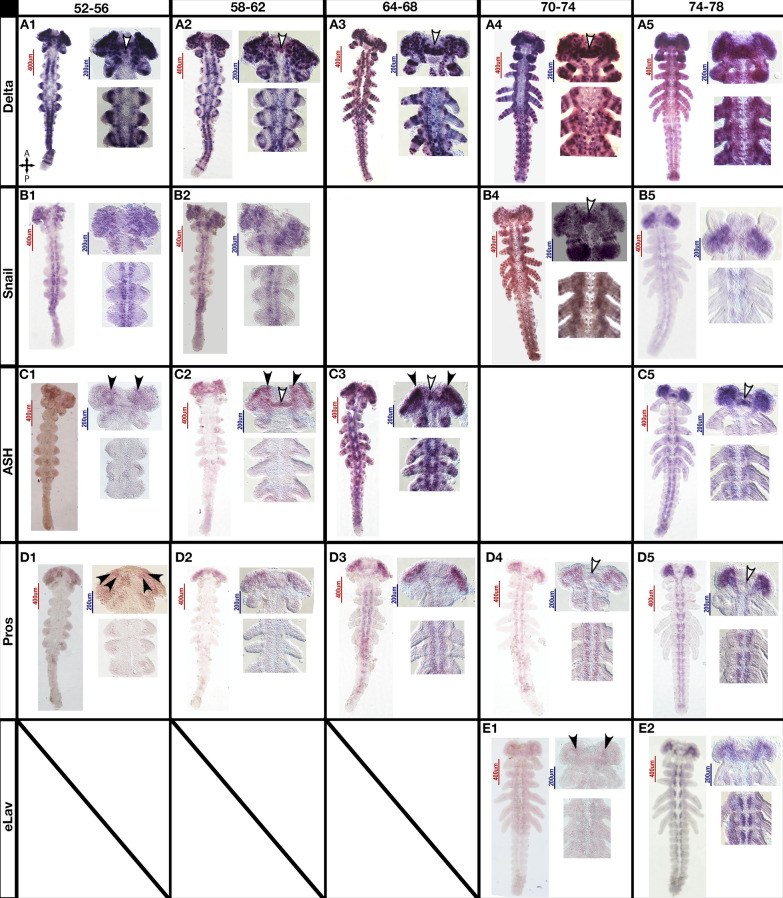
Expression of pro-neural and neuronal genes during mid to late germband in of *Oncopeltus fasciatus*. A timed series of mRNA in-situ hybridization during mid to late germband formation stage in *Oncopeltus*. The columns show embryos of the same age, and the rows show embryos stained for the same gene. Anterior towards the top. The same regions are shown in each series, in the same order, although magnified regions are not always from the same specimen: the entire germband, a higher magnification image of the head lobes, and a higher magnification image of thoracic segments 1–3. *Delta* expression marks cells with neurogenic potential and neuroblasts. *Snail* marks asymmetrically dividing and migrating neuroblasts. *ash* marks proliferating neuroblasts, *pros* marks differentiating neuroblasts, and *elav* marks neurons. Scale bars for full embryos are 400 μm, and for magnified regions 200 μm

**Fig. 6:**
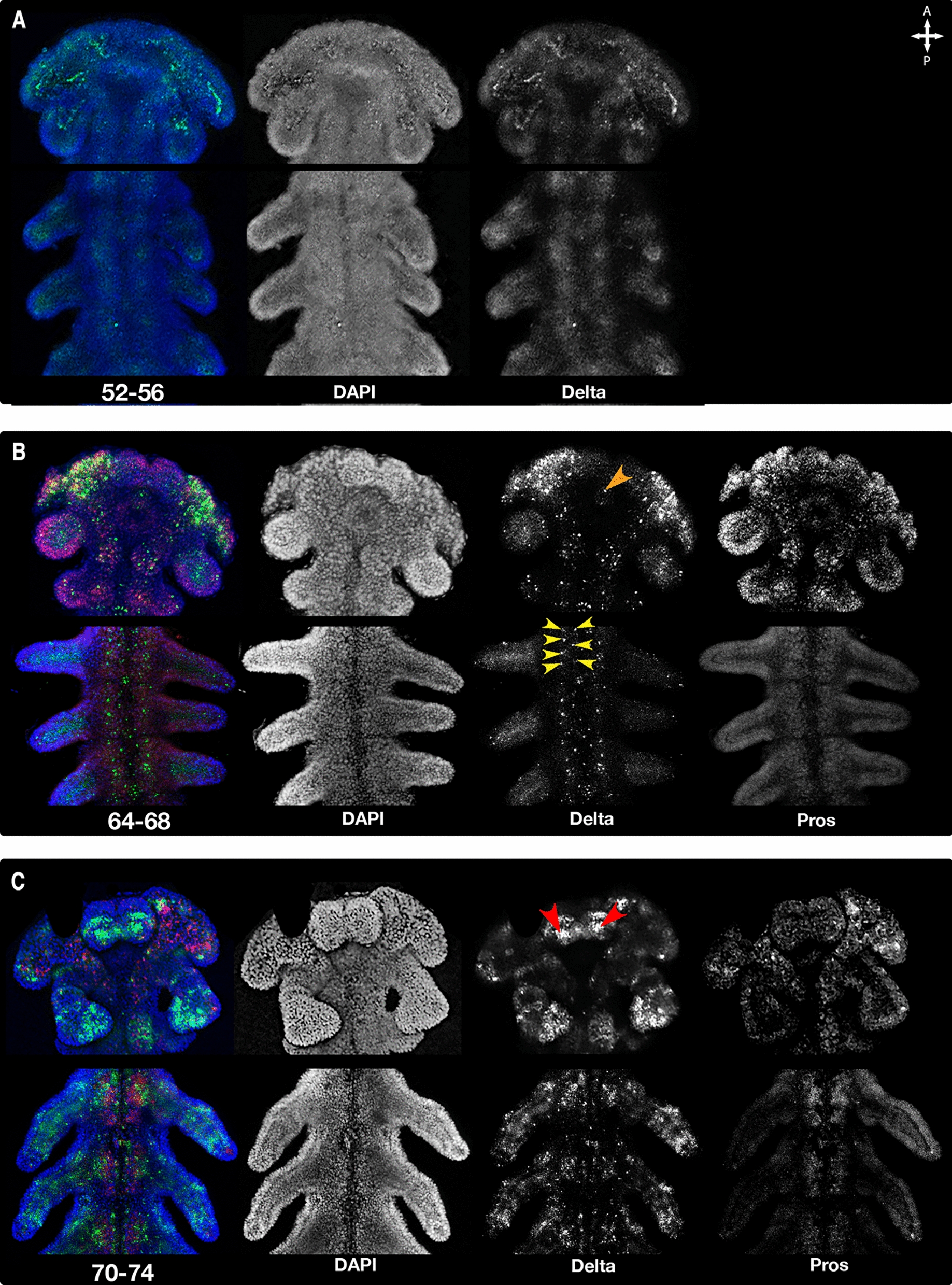
Expression of *Delta* and *pros* during the mid to late germband stage. Merged channels on the left—DAPI in blue, *Delta* in green, *pros* in magenta, followed by individual channels in greyscale. Anterior towards the top. **A** *Delta* expression starts weakly in a broad area, however even at this stage expression is stronger in the head, marking cells with neurogenic potential, that appear to cluster. **B** *Delta* expression focuses to single cells (green dots) and seems to mark cells that develop into neuroblasts, as they delaminate into the embryo and divide multiple times into daughter cells expressing *pros* (red dots) but not *Delta* (no co-expression of green and magenta- no yellow dots). By the end of segmentation **C** there is a robust population of *pros*-expressing cells (red dots) in the central nervous system, while some of the *Delta* expressing cells have disappeared, leaving behind the daughter cell population

The earliest pro-neural marker that we used was *Delta,* as it marks cells with neuronal potential, but with a stronger expression in the selected neuroblast within each pro-neural cluster (Fig. 5, A1, arrowhead). Since *asense* does not exist in Hemiptera [35, 36] (Supplementary Fig. 1), pro-neural clusters are probably marked by *Delta*, as its expression appears in a dotted pattern both in the head and in the thorax. In the segmenting abdomen, marked pro-neural clusters appear sequentially after the emergence of each segment, showing a switch in *Delta* gene function from its segmentation role (Fig. 5, A2–A4). This can be seen as the pattern transforms from segmental stripes into dots in the posterior of the trunk. The expression of *Delta* (Fig. 5, A1–A4) begins diffusely and then localizes to single cells, including neuroblasts in each hemi-segment and a single neuroblast between segments. In each hemi-segment we can see between 6–7 neuroblasts (Fig. 6B, yellow arrowheads); Based on this we suggest that *Delta* marks the pro-neural clusters simultaneously in the head and thoracic segments and sequentially in the abdominal segments.

*Snail* marks future neuroblasts (Fig. 5, B1–B2), indicating delamination and asymmetrical division (Fig. 5, B1–B5) [27], and confirms the selection of neuroblasts within pro-neural clusters. The expression of *snail* (Fig. 5, B3–B4), hints at cell movements at the beginning of organogenesis in the trunk, as its known roles in other insects also include cell movement and asymmetrical division [27, 28]. Its diminished expression in the trunk compared to the head during late stages of segmentation (Fig. 5, B5), might suggest that while most neuroblasts have already finished their role in the trunk, some remain active in the brain lobes, marking a longer period of neurogenesis inside the brain lobes.

In many insects, *achaete-scute* complex genes are found in pro-neural clusters [13, 14]. *O. fasciatus* has only a single copy of a single gene from this family- *ash.* Surprisingly,* ash* expression was not found in the trunk during early neurogenesis (Fig. 5, C1–C2). Based on this, it would appear that *ash* does not mark pro-neural clusters in *Oncopeltus*. The expression *of ash* starts in the PGS (Fig. 5, C1–C5) and remains exclusive to it until around 64–68 hAEL. No earlier expression of *ash* was found, supporting the hypothesis that the single copy of *ash* in *O. fasciatus* only functions as a maturation signal, as it has no earlier expression in the pro-neural clusters. Activation of *ash* in the trunk occurs simultaneously in the gnathal segments and the thorax, and sequentially in the abdomen, matching the mode of segmentation that gave rise to these segments. The first cells marked for neural fate appear in the brain lobes (Fig. 5, C2), followed slightly later by the segments of the gnathal region and the thorax (Fig. 5, C3).

*Pros*, like *ash,* is not expressed in the trunk during early neurogenesis; its expression beginning in the PGS (Fig. 5, D1–D2). Large cells are visible at the median parts of the brain lobe. Around 64–68 hAEL, *pros* is expressed simultaneously in the gnathal segments and in the thorax, and sequentially in the abdomen. The medial ganglion mother cells – recognized by expression of *pros* and their smaller size compared to the neuroblasts marked by *delta* and *snail* at the same age (Fig. 5, A4–B4)—appear shortly after the appearance of early neuroblasts in each segment (Fig. 5, A3–D3, A4–D4, , supplementary Fig. 5A). The same switch in cell differentiation occurs along the entire trunk, regardless of segment age, in the simultaneously segmenting parts (gnathal segments and thorax) and sequentially in the abdomen.

*Elav* marks newborn neurons (Fig. 5E), and is only expressed from 70 hAEL on. This expression starts simultaneously in both head and thorax, (Fig. 5, E1) but is shortly followed by expression in the whole embryo (Fig. 5, E2).

### Neural differentiation in the brain

Neurogenesis in the PGS begins earlier (as seen by early onset of expression of neurogenesis genes) and progresses faster than in the rest of the embryo, but otherwise follows a similar trajectory. Expression of *snail* in the nervous system starts at 52–56 hAEL (Fig. 5, B1). This expression is both in brain and in the trunk. However, expression of *ash* and *pros* are limited only to a specific sub-population of brain neuroblasts at this stage. This brain-specific expression begins with four neuroblasts that appear bilaterally at an anterior-medial position in the brain lobes (Fig. 5C–D, arrowheads. Supplementary Fig. 5A). The median parts of the brain lobes and a medial neuroblast (white arrowhead, Fig. 5, A2, C2, Supplementary Fig. 5A) start expressing *ash* and *pros* a bit later and are therefore assumed to be younger than other cells in this region (Fig. 5C–D).

Based on its location and structure, we suggest that the diffuse, bridge-like *Delta* signal in the PGS during early-middle germband stage (52–56 hAEL) (Fig. 5, A1–3) indicates the future location of the brain commissure or other neuronal structures, based on the anatomy of the hatchling brain (34). This can be seen more clearly in the HCR staining (Fig. 6A), where the signal is initially diffuse, and later (Fig. 6B, orange arrowhead) becomes localized to individual cells.

Structurally, it can be seen that the neuroblasts delaminate into the embryo starting at 68 hAEL or earlier (Fig. 3B) and that most neuroblasts are separated from the ectoderm by 74 hAEL (Fig. 3D) and fully separated by 80hAEL (Fig. 3F). It can be seen that although with time, the neuroblast population, marked by *Delta*, is depleted, it does not fully disappear in the analyzed time frame (Fig. 5, A–C5). New neuroblast populations continues to appear, mostly ones presumably whose progeny belongs to the peripheral nervous system (e.g. in the labium, Fig. 6C Red arrowheads).

## Discussion

### Overview of *oOncopeltus* neurogenesis

**Fig. 7:**
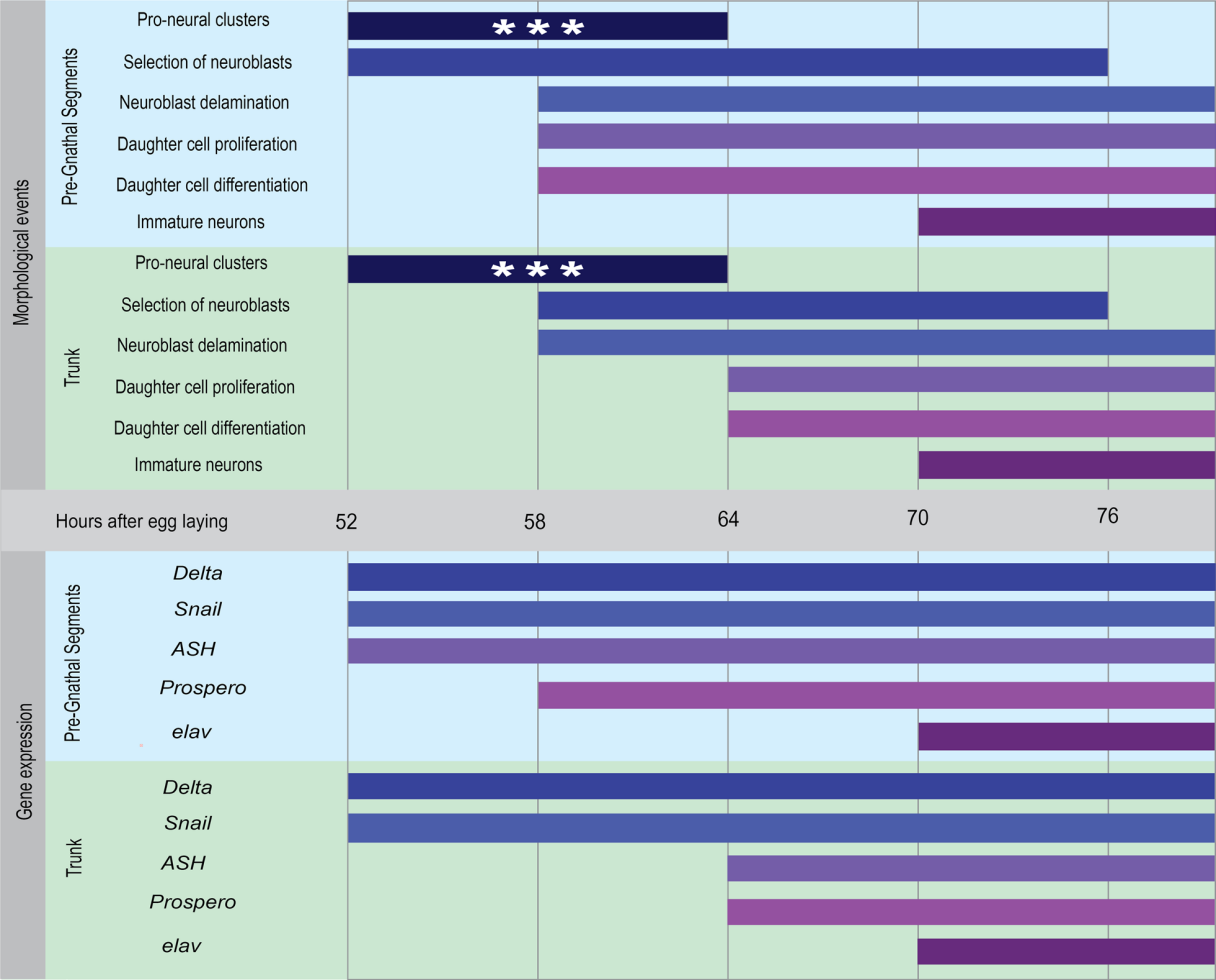
Summary of morphological events and gene expression during neurogenesis in *O. fasciatus*. The top panel shows the progression of morphological milestones, while the bottom shows the expression of the canonical pro-neural and neuronal developmental marker genes. The blue and green sections within each panel show the difference in timing between the PGS and the trunk. White asterisks mark a suggestion not currently supported by our data

Neuroblasts, GMCs and their progeny appear in a symmetrical, stereotypical pattern that is repeated between segments as in all previously described insects. This is also true for the *O. fasciatus* embryo, starting from mid-germband formation stages. The first phase of neurogenesis starts in the PGS, and a second one occurs slightly later, in the entire body (Fig. 7).

There are earlier born neuroblasts (Fig. 3, A1–B2), which can be seen expressing *Delta* in the PGS by 58–62 hAEL (Fig. 5A, B). We suggest that these cells are likely to be type II neuroblasts, similar to what has been described in *Tribolium castaneum* and *Schistocerca gregaria* [8, 16, 31, 41] as their size and location match previous descriptions. However, further examination is needed to validate the gene expression profile of this cell population. In addition, a single large cluster can be seen in each of the lobes (Fig. 6C, orange arrowhead).

It is worth pointing out the existence of an arch of *Delta* expression connecting the two brain lobes (Fig. 5, A1–A3—white arrowhead). The center of *Delta* expression marks the spot in which a single medial neuroblast will appear in the head (Fig. 6B, orange arrow). This unpaired *Delta* expression pattern is not seen anywhere else in the embryo. Based on the anatomy of the hatchling *O. fasciatus* brain [34], we suggest this marks the location of the future brain commissure.

In the trunk, between six and seven neuroblasts can be seen in each hemi-segment (Fig. 6B, yellow arrowheads), by late-stage segmentation (66–68 hAEL). This is very similar to the pattern of neurogenesis in the trunk of other insects [5, 9, 13, 14].

The segmental ganglia can be seen forming all along the embryo by the end of segmentation (Fig. 5, 70–74 hAEL, 74–78 hAEL). However, by the time of hatching, the only ganglia seen in the larva are the suboesophageal ganglion ventral to the brain, and the thoracic ganglia [34]. The central nervous system represents a smaller proportion of the total body mass of the adult *O. fasciatus* compared to its proportion in the first instar larva [34]. Data from other insects with similar nervous systems in which thoracic ganglia have fused [3, 9] support the idea that most of these cells migrate rather than undergo cell death.

### *Oncopeltus* does not have *achaete-scute* complex gene expression in pro-neural clusters

We have not found early *ash* expression in *O. fasciatus* (Fig. 5C). Instead, *Delta* appears to mark either a small group of cells early on (Fig. 5A) or individual cells (Fig. 5B). This expression is the closest to what we would expect to see for pro-neural clusters*.* This is quite unexpected, as previous data from other insects suggests that bHLH genes, particularly *asense* and *ash*, select ectodermal cells for neural fate [2, 42, 43]. The insect model suggests that *Delta* expression progresses via lateral inhibition into being limited to a single neural progenitor selected from the cluster. The lateral inhibition driving this process is often facilitated by bHLH genes [7, 22, 26]. However, this appears not to be the case in *O. fasciatus,* and may not be the case in other members of Hemiptera that also lack *asense* [35, 36] and only have a single copy of *ash*. So, while *Delta* appears to be laterally inhibited, there is no regulatory input from *ash*, as this gene is not expressed in the pro-neural clusters of *O. fasciatus*. We suggest there is another mechanism that leads to the selection of neuroblasts within the pro-neural cluster. This might also be the case for other hemipterans missing the *asense* gene*.* Since this gene family has a complex history in insects [35, 36, 42, 43], involving several cases of duplication and splitting, while gaining and losing different members (see supplementary Fig. 1), it seems that the role *ash* plays in the pro-neural clusters might not be ancestral in insects. Nonetheless, we cannot rule out the possibility that in *O. fasciatus*, a different currently unknown bHLH gene might have taken on the role of selecting neural precursors.

This find raises an intriguing possibility, which needs to be followed up with additional data, that *O. fasciatus* and other hemipterans may have pro-neural clusters that function very differently from well-studied holometabolous models. If true, this would mean that at least some hemimetabolous insects maintain a situation similar to the plesiomorphic condition seen in myriapods and chelicerates [21, 28, 44–46]. A more detailed future analysis can teach us about the evolution of the process of neuroblast selection, particularly about the role of other genes and ligands that may be drivers of lateral inhibition, as well as other possible signaling modes that enable selection of specific cells for neurogenic fate out of a cluster.

### Dynamics of neurogenesis matches the dynamics of segmentation

An intriguing, though perhaps unsurprising, finding from our analysis is the similarity between the dynamics of segmentation and neurogenesis. In regions where segmentation occurs simultaneously (e.g. the thoracic and gnathal segments), neurogenesis also progresses simultaneously across all segments. In contrast, in the abdomen, where segmentation is sequential, neurogenesis proceeds sequentially from anterior to posterior. This suggests that the age of segments within the trunk plays a crucial role in the progression of neurogenesis. It is also worth noting that neurogenesis occurs earlier and more rapidly in the pre-gnathal segments, where the brain will form, compared to the trunk (Fig. 7).

Many genes are involved in both segmentation and neurogenesis [47–50]. However, the connection between these processes remains unclear. We propose that the similarity in the dynamics of segmentation and neurogenesis provides an opportunity to explore the link between the two. The most promising candidate networks for this investigation are the neuroblast timer and gap gene networks, as they share several commonalities [50]. Notably, the genes *Krüppel* and *hunchback* are the first genes in both networks and play roles in both neurogenesis and segmentation in myriapods, chelicerates and insects [46, 48–50], and even in non-arthropod species [51, 52]. In these networks, and other genes involved in both segmentation and neurogenesis (e.g., *hedgehog* and *engrailed*) [47, 49, 51], we observe a shift from a role in segmentation and body patterning during early development to a role in neurogenesis during later development. This shift is perhaps most evident in the expression of *Delta* in our current data. These findings support the idea that many genes were co-opted for segmentation roles after the emergence of nervous systems, potentially in a convergent manner across different lineages [5, 51, 52].

### Brain development follows a different trajectory relative to trunk neurogenesis

The very first proliferating and differentiating neuroblasts appear in the brain lobes, followed shortly afterwards by neuroblasts at the base of the antennal segment and the intercalary segment. Neuroblasts appear in the trunk segments only later (Fig. 5). The boundary between the PGS and the trunk is very distinct both in terms of gene expression and in the morphological progress of neurogenesis, hinting that spatial cues, and not only temporal cues are behind the difference between the trunk and PGS.

The appearance of the younger neuroblast population in the PGS while neural precursors start proliferating in the trunk supports the idea of more than one initiation signal for cell proliferation in *Oncopeltus* (Fig. 5): one, unique to the PGS that initiates development only in the PGS, and a later one that initiates a whole-embryo neurogenesis process. The two populations are selected at the same time, as *Delta*, the best marker we have for the selected neuroblasts, appears in the whole embryo simultaneously, but their proliferation is initiated by at least two different signals, as evident from *ash* and *pros* expression. The first phase affects the PGS only, while the second phase affects both PGS and trunk, as far as we can tell from our current data (Fig. 5), leading to two distinct populations of neuroblasts in the PGS: early differentiating, and later differentiating.

The idea of two different neuroblast populations is supported by the α-tubulin antibody staining that shows a mass of cells inside the brain lobes, creating the significant thickening of the head lobes at this stage and also the distinct folding that brings lateral parts of the head into a more central position. Not only does morphogenesis start earlier in the PGS compared to the trunk, but there is more cell division occurring inside the brain lobes compared to the surface of the embryo (Fig. 4). Gene expression also supports this, as all markers checked keep being strongly expressed in the PGS (Fig. 5). In addition, it also appears that both proliferation and differentiation of neural cells continue in the PGS even past the loss of neuroectoderm in the trunk, and the differentiation of young neurons (Fig. 3F). This can be seen inside the brain lobes, as they begin to undergo morphogenesis to form the brain, a process that extends beyond the scope of the current study.

### Concluding remarks

While neurogenesis in Holometabola is well understood, focusing on holometabolan insects leads to a bias in the way we think about insect neurogenesis. Genes involved in the process have duplicated, specialized and changed dramatically to allow for very rapid neurogenesis, functioning mainly in two waves- once during embryogenesis and a second during metamorphosis. To properly understand the ancestral roles of the genes and the neurogenesis process in insects, we must learn more about the way it functions outside of Holometabola.

## Supplementary Information

Additional file1 (PNG 758 KB)

Additional file2 (PNG 7361 KB)

Additional file3 (PNG 9798 KB)

Additional file4 (PNG 7567 KB)

Additional file5 (PNG 4754 KB)

Additional file6 (PNG 4848 KB)

Additional file7 (PNG 608 KB)

Additional file8 (PNG 2123 KB)

Additional file9 (PNG 145 KB)

Additional file10 (PNG 18174 KB)

Additional file11 (DOCX 81 KB)

## Data Availability

All relevant data are included within the manuscript.
