# Supplementary figures and images for: Neurogenesis in the trunk and brain of the milkweed bug Oncopeltus fasciatus: insights beyond holometabolan models
Source: Front Zool. 2025 Dec 10;23:3. doi: 10.1186/s12983-025-00593-z (PMC12821930; doi:10.1186/s12983-025-00593-z)

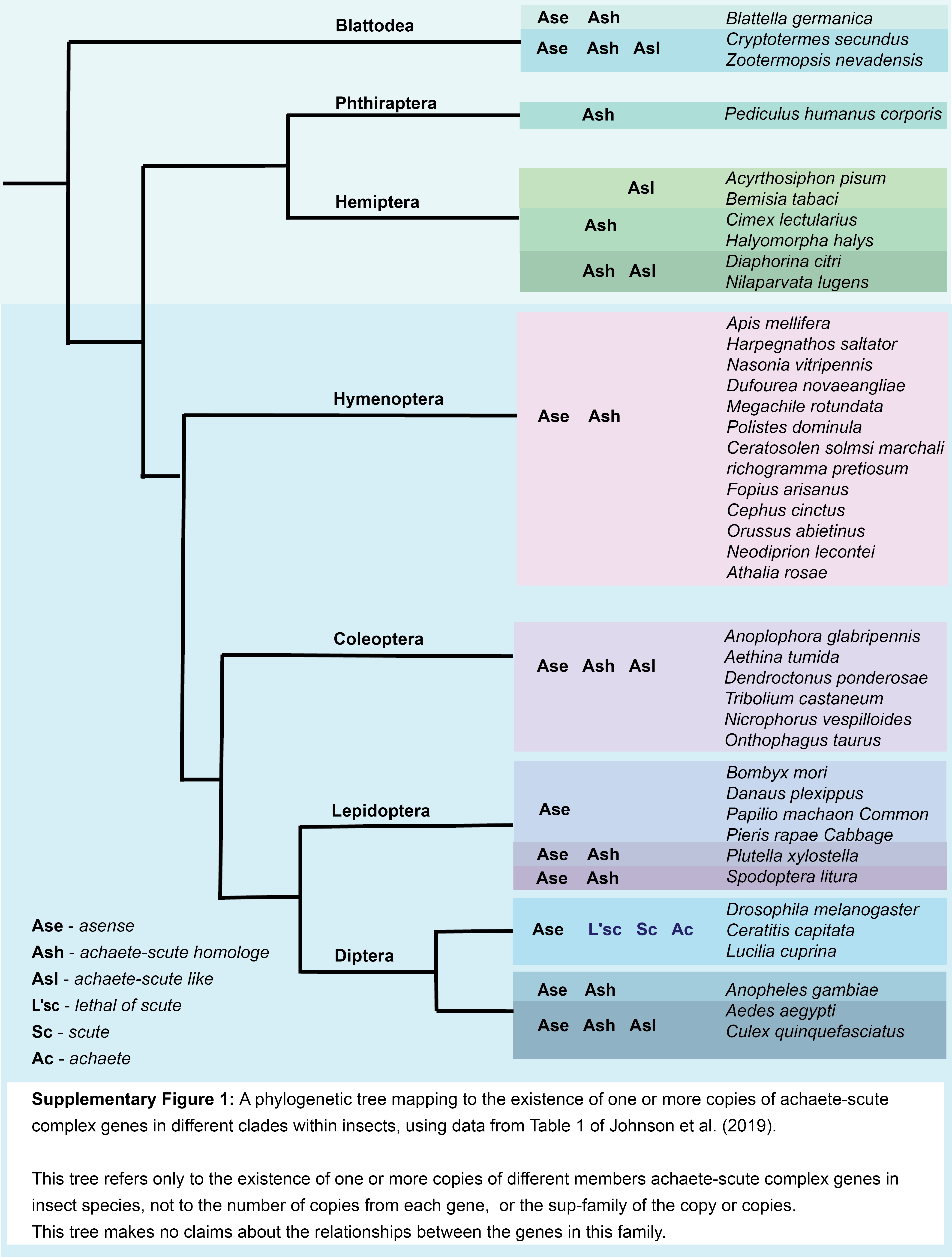

Supplement: Supplementary file 1 — Additional file1 (PNG 758 KB) [file 12983_2025_593_MOESM1_ESM.png]

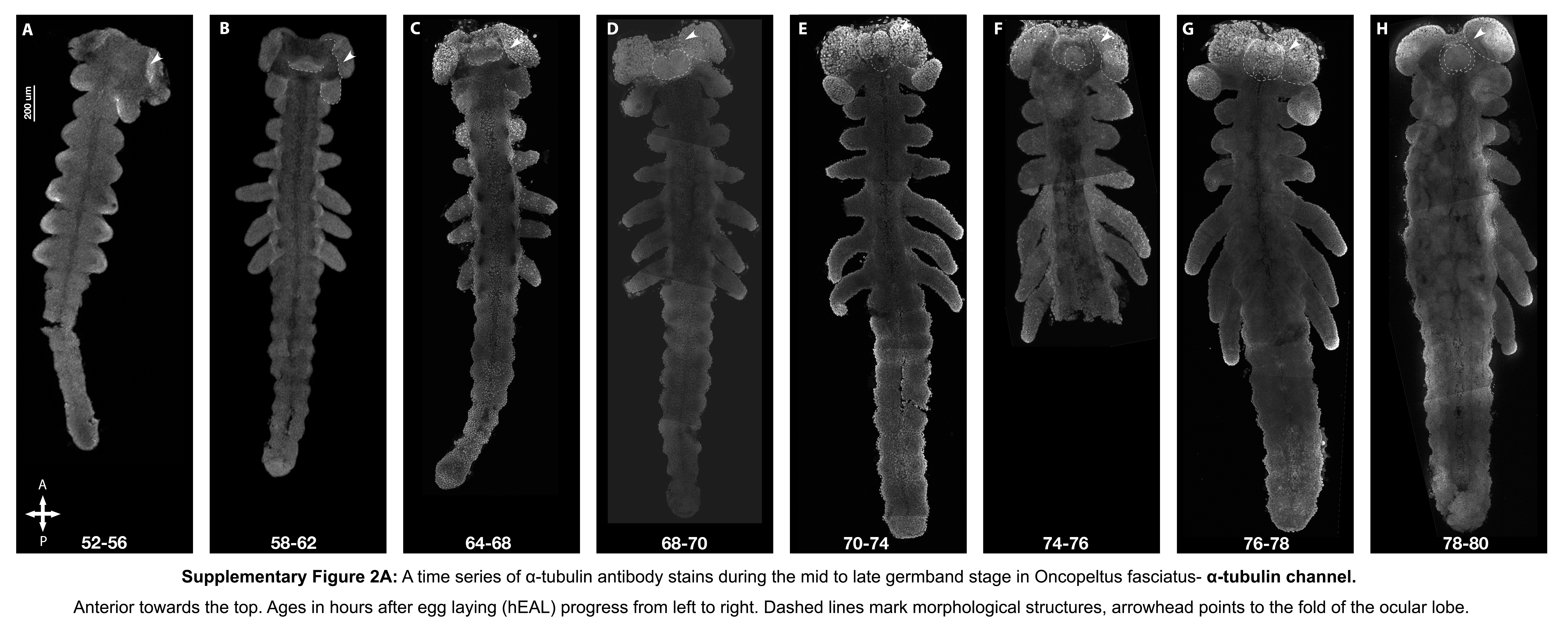

Supplement: Supplementary file 2 — Additional file2 (PNG 7361 KB) [file 12983_2025_593_MOESM2_ESM.png]

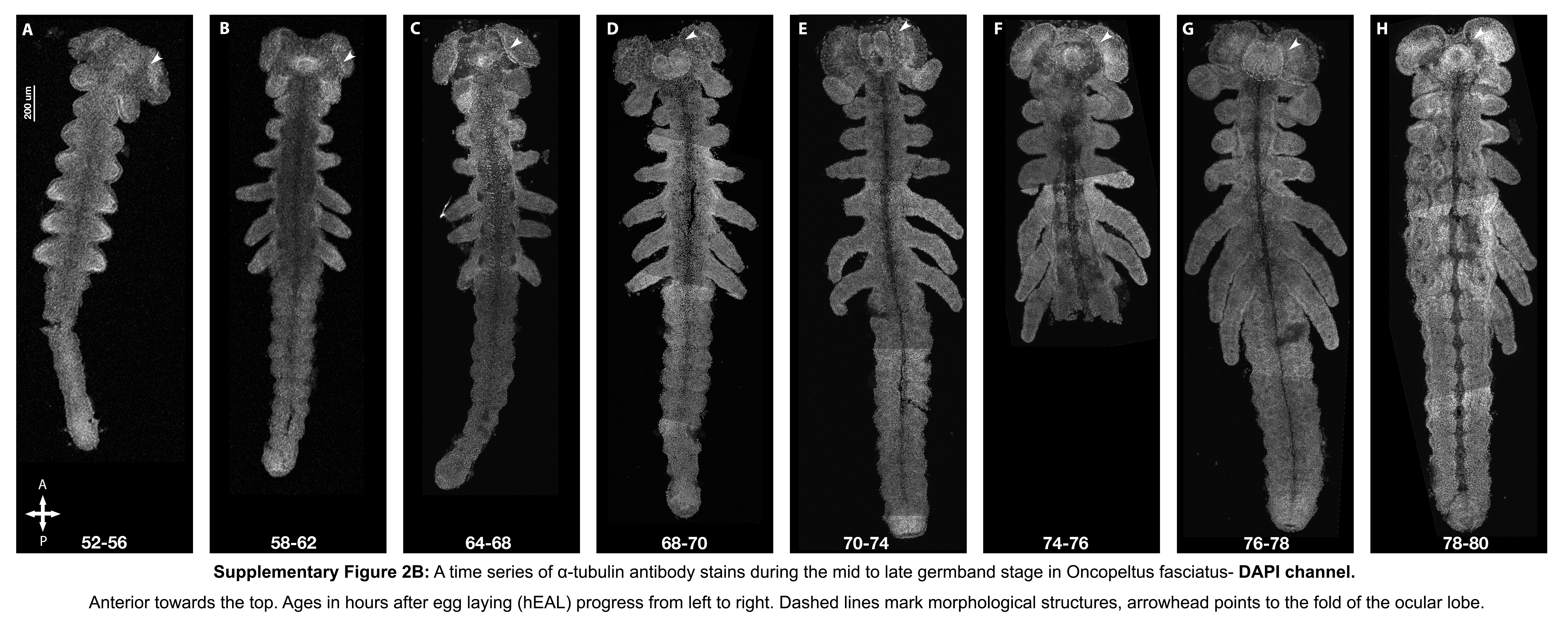

Supplement: Supplementary file 3 — Additional file3 (PNG 9798 KB) [file 12983_2025_593_MOESM3_ESM.png]

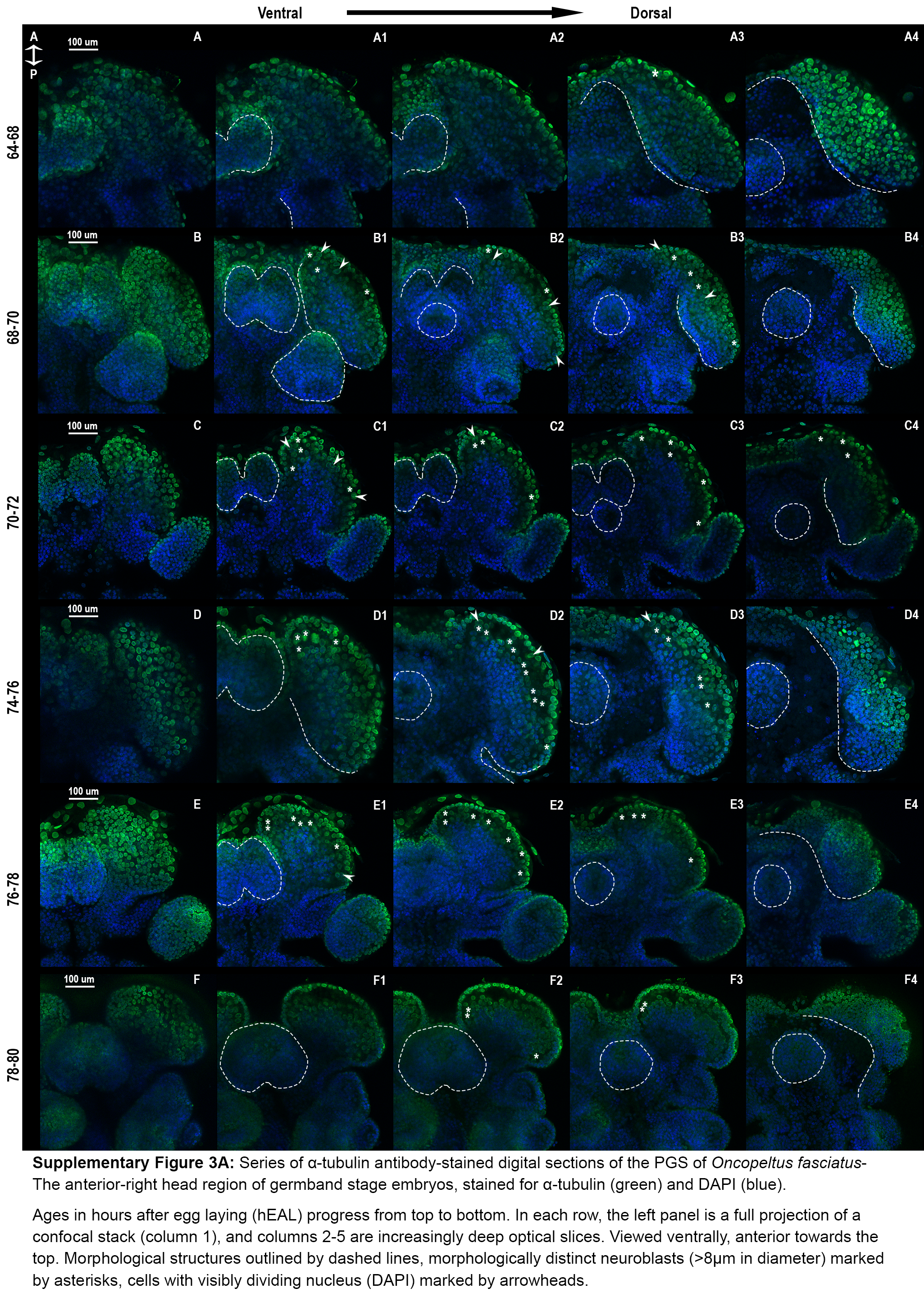

Supplement: Supplementary file 4 — Additional file4 (PNG 7567 KB) [file 12983_2025_593_MOESM4_ESM.png]

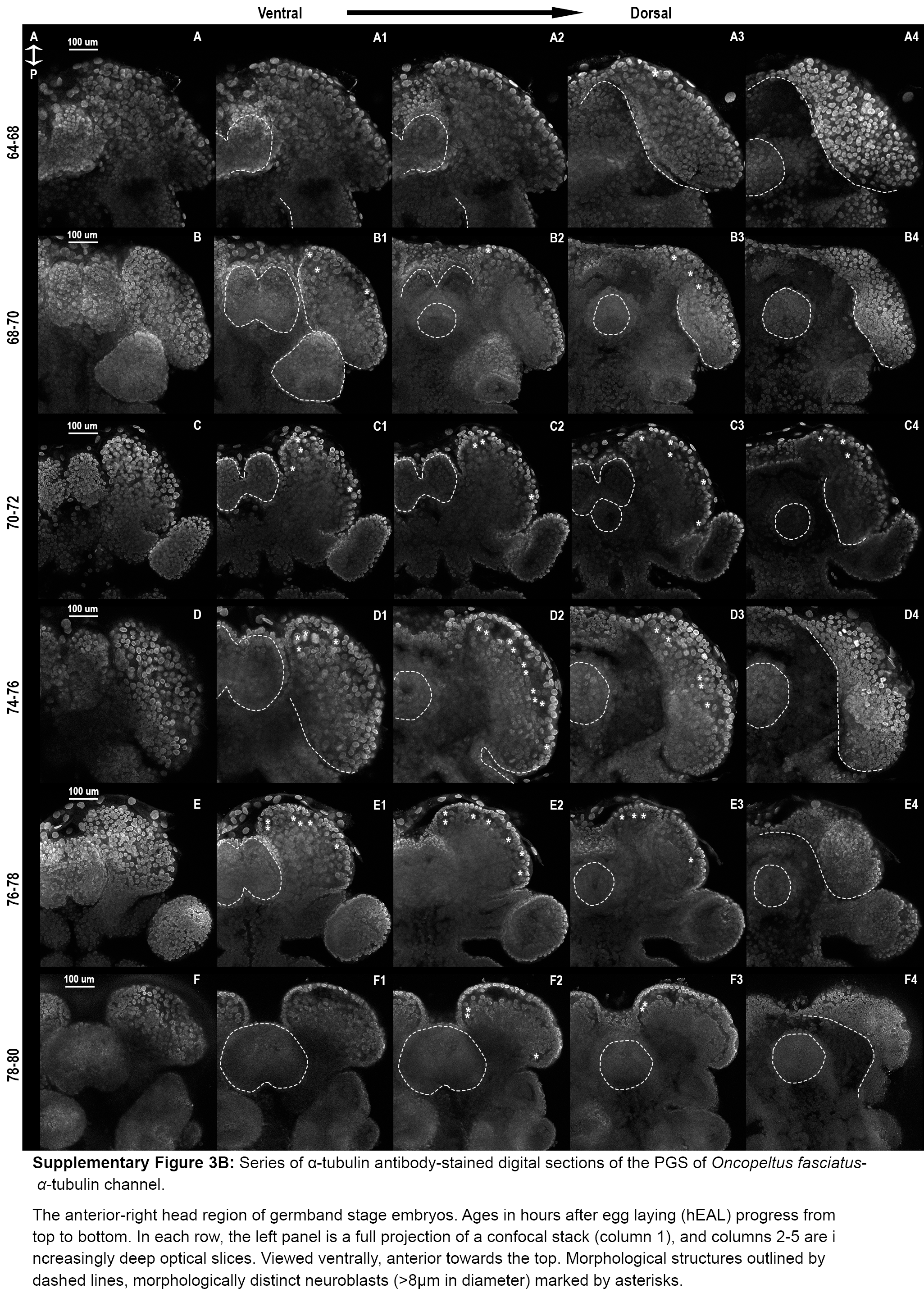

Supplement: Supplementary file 5 — Additional file5 (PNG 4754 KB) [file 12983_2025_593_MOESM5_ESM.png]

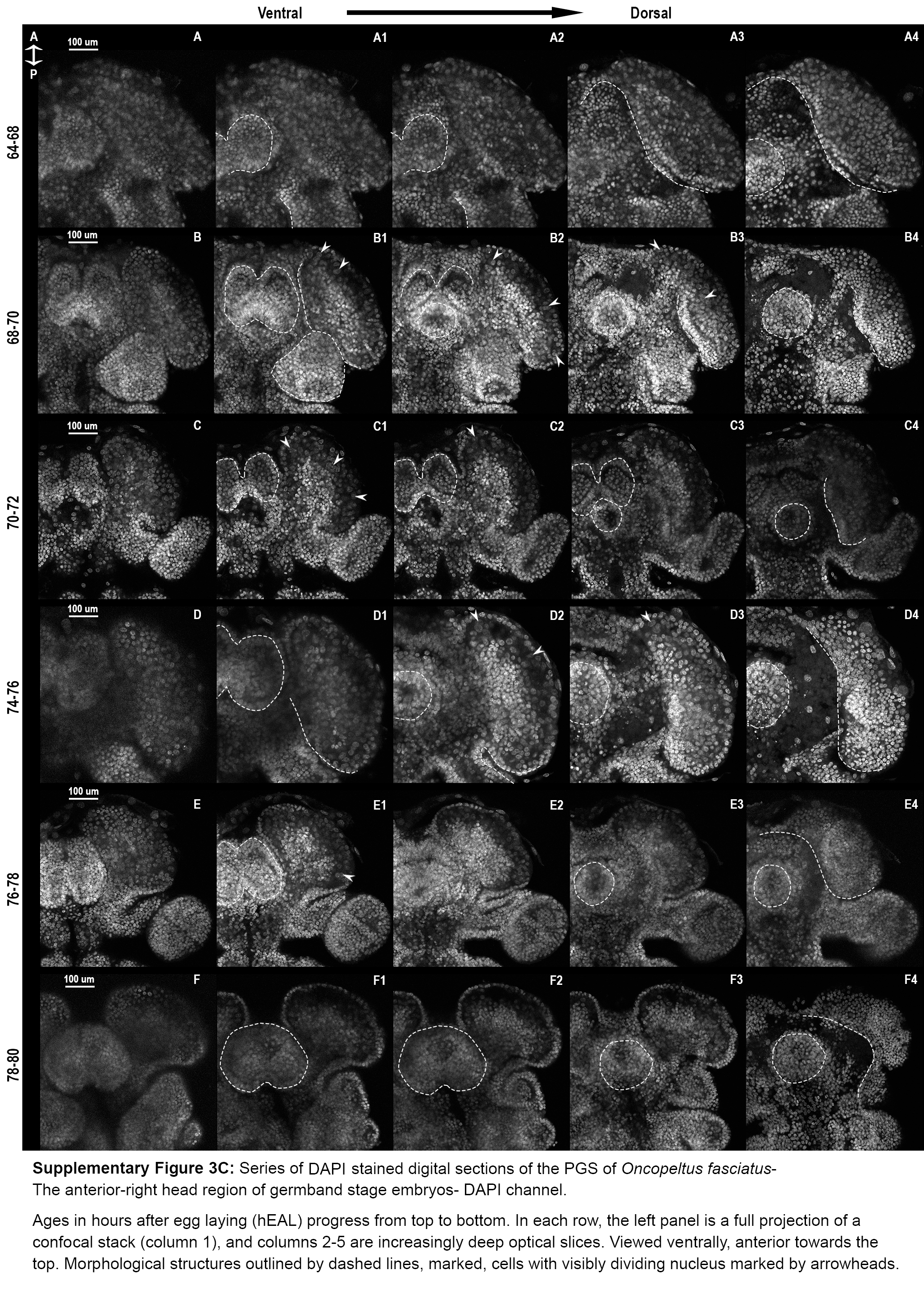

Supplement: Supplementary file 6 — Additional file6 (PNG 4848 KB) [file 12983_2025_593_MOESM6_ESM.png]

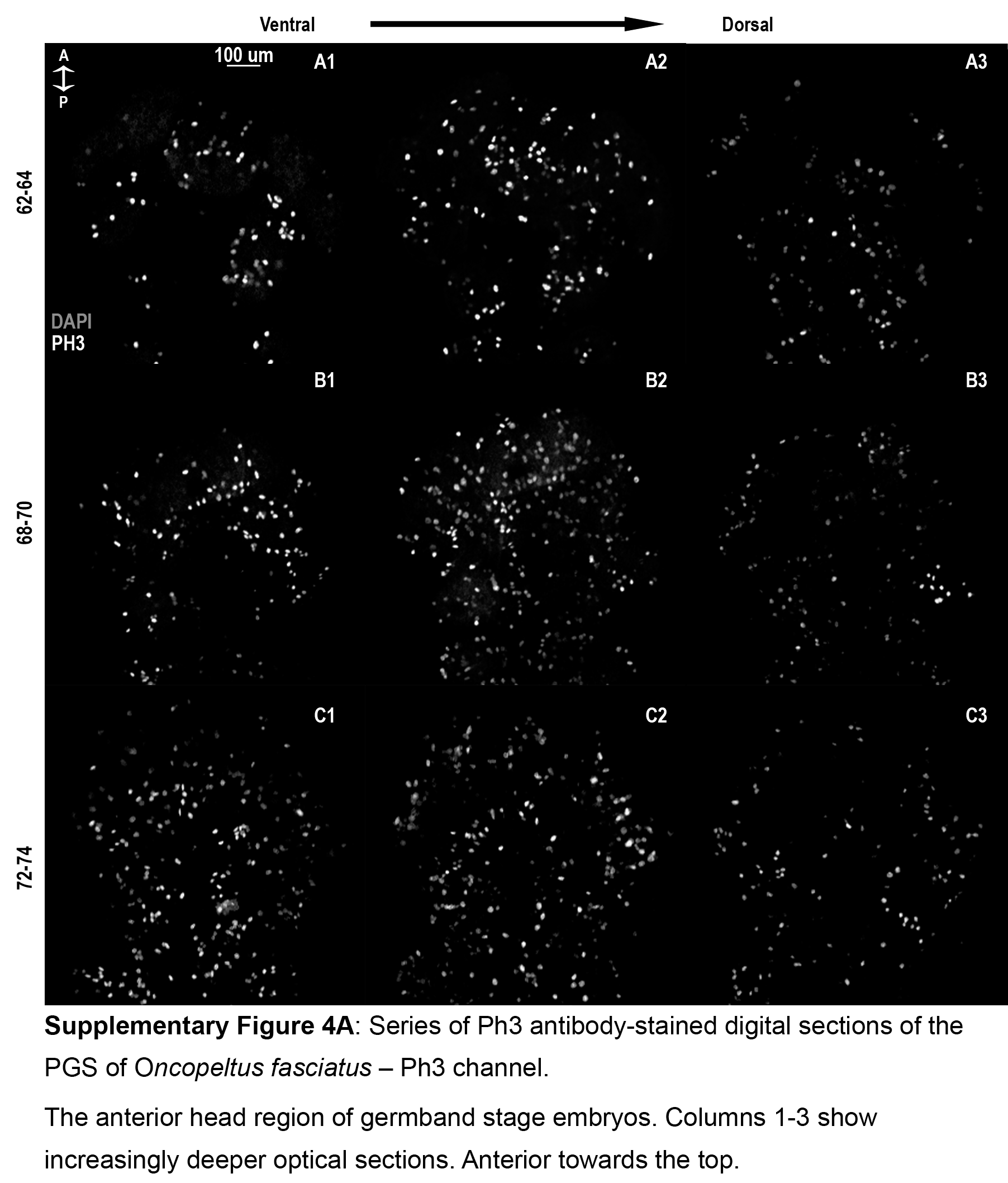

Supplement: Supplementary file 7 — Additional file7 (PNG 608 KB) [file 12983_2025_593_MOESM7_ESM.png]

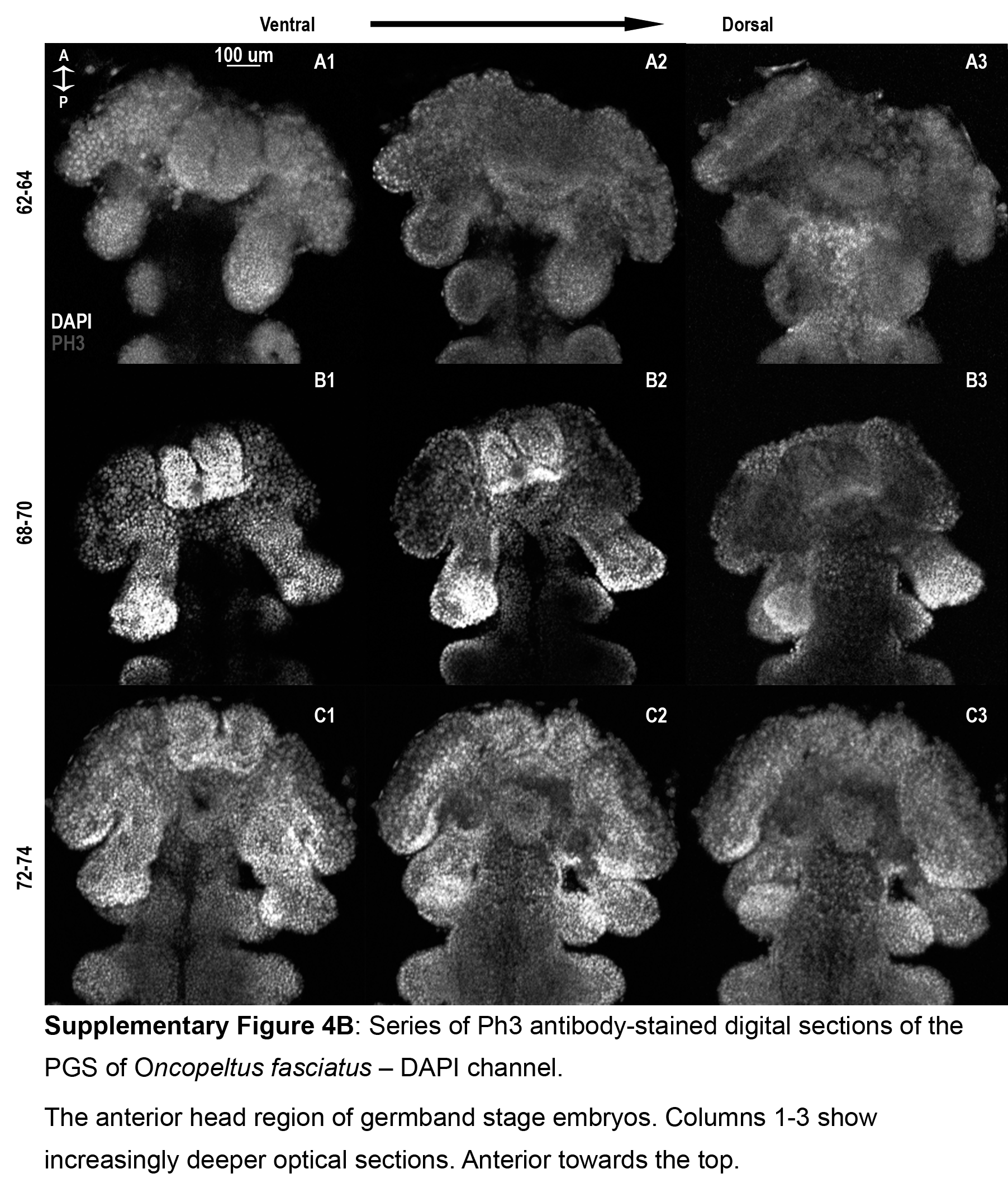

Supplement: Supplementary file 8 — Additional file8 (PNG 2123 KB) [file 12983_2025_593_MOESM8_ESM.png]

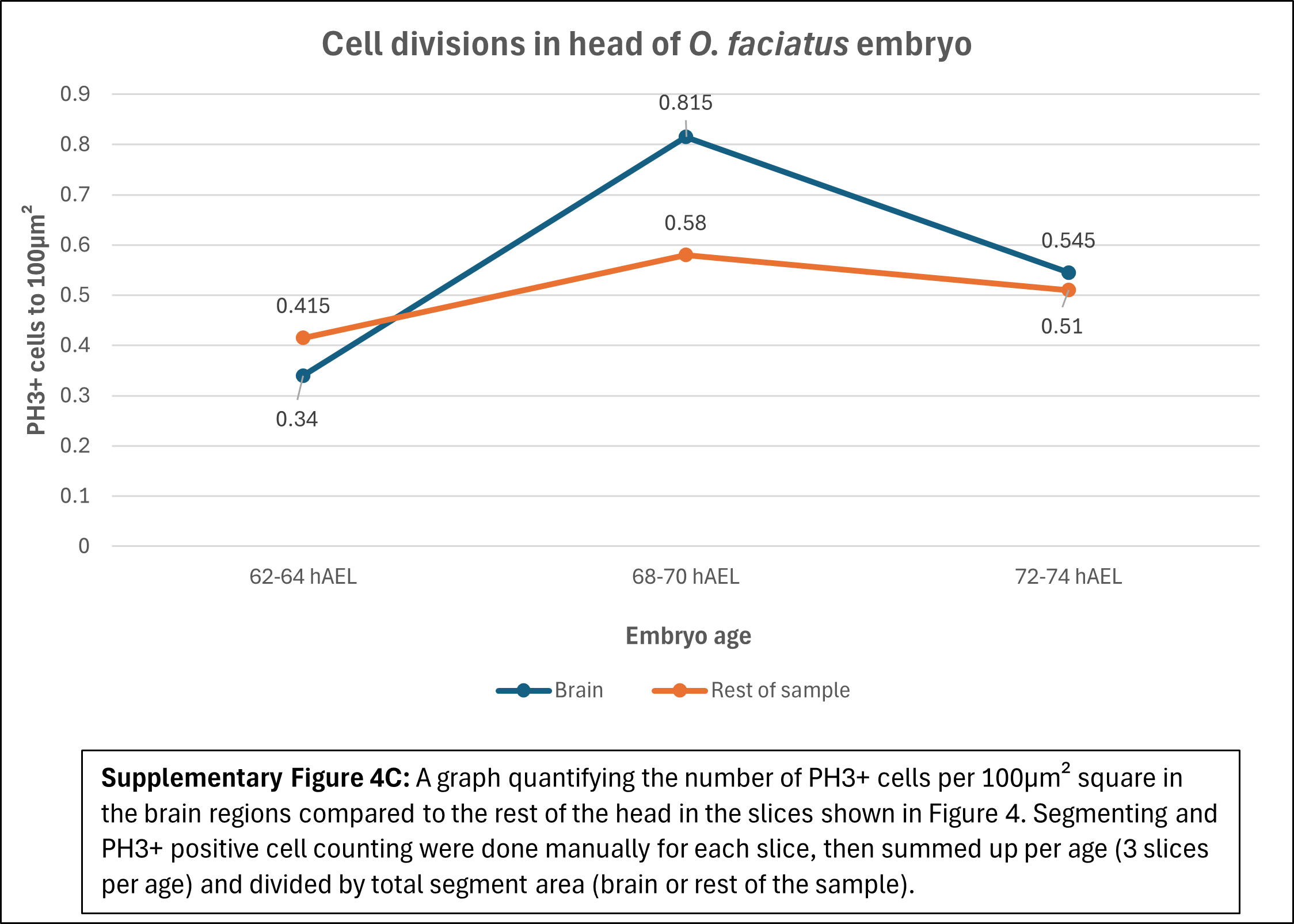

Supplement: Supplementary file 9 — Additional file9 (PNG 145 KB) [file 12983_2025_593_MOESM9_ESM.png]

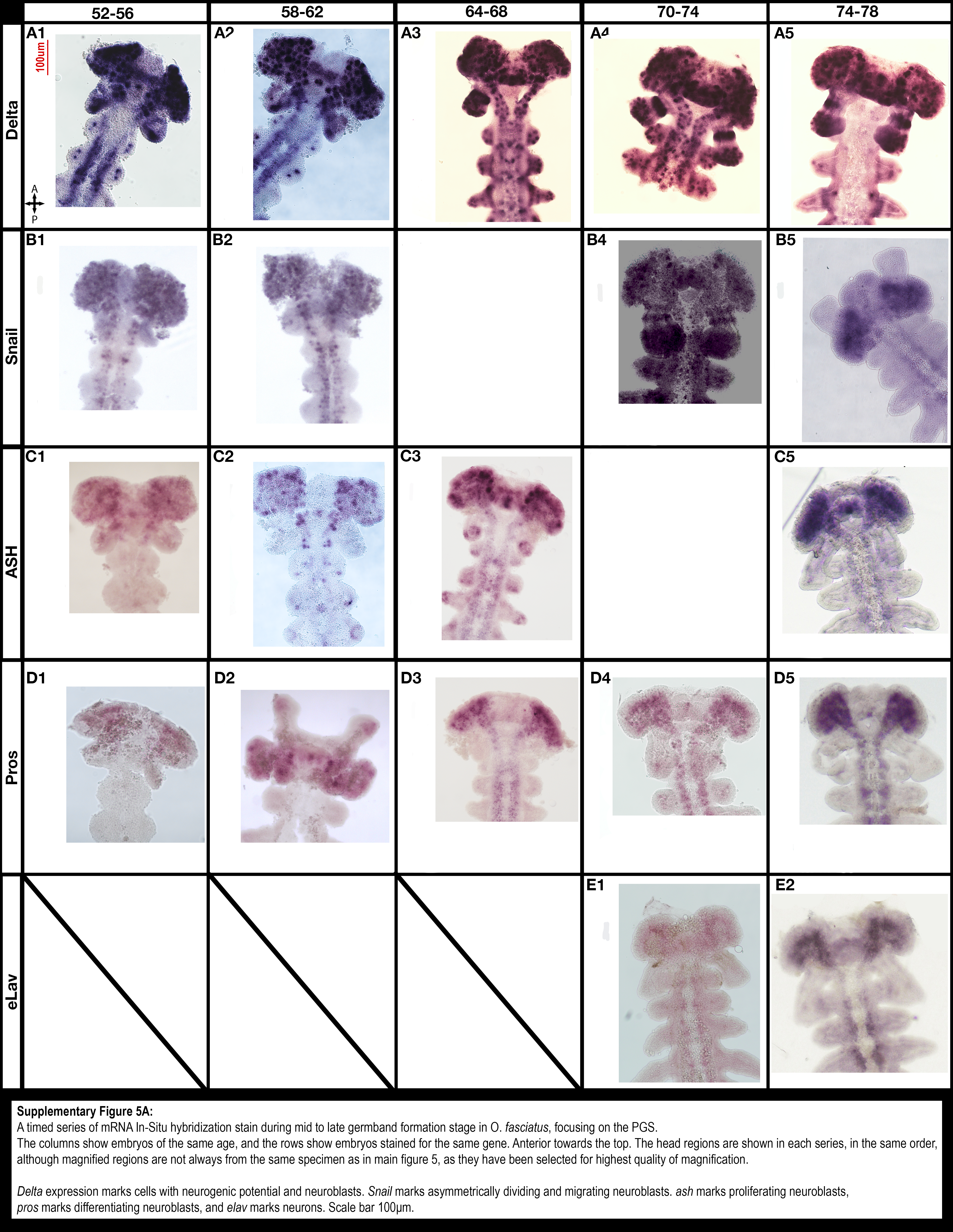

Supplement: Supplementary file 10 — Additional file10 (PNG 18174 KB) [file 12983_2025_593_MOESM10_ESM.png]
