# Supplementary material for: Neurogenesis in the trunk and brain of the milkweed bug Oncopeltus fasciatus: insights beyond holometabolan models
Source: Front Zool. 2025 Dec 10;23:3. doi: 10.1186/s12983-025-00593-z (PMC12821930; doi:10.1186/s12983-025-00593-z)
Supplement: Supplementary file 11 — Additional file11 (DOCX 81 KB) [file 12983_2025_593_MOESM11_ESM.docx]

**Supplementary Figure 1:** A phylogenetic tree mapping to the existence of one or more copies of achaete-scute complex genes in different clades within insects, using data from Table 1 of Johnson et al. (2019). This tree refers only to the existence of one or more copies of different members achaete-scute complex genes in insect species, not to the number of copies from each gene, or the sup-family of the copy or copies. This tree makes no claims about the relationships between the genes in this family.

**Ase -** *asense*

**Ash -** *achaete-scute homologe*

**Asl -** *achaete-scute like*

**L'sc -** *lethal of scute*

**Sc -** *scute*

**Ac -** *achaete*

**Supplementary Figure 2A:** A time series of α-tubulin antibody stains during the mid to late germband stage in Oncopeltus fasciatus- α-tubulin channel. Anterior towards the top. Ages in hours after egg laying (hEAL) progress from left to right. Dashed lines mark morphological structures, arrowhead points to the fold of the ocular lobe.

**Supplementary Figure 2B:** A time series of α-tubulin antibody stains during the mid to late germband stage in Oncopeltus fasciatus- DAPI channel. Anterior towards the top. Ages in hours after egg laying (hEAL) progress from left to right Dashed lines mark morphological structures, arrowhead points to the fold of the ocular lobe.

**Supplementary Figure 3A:** Series of α-tubulin antibody-stained digital sections of the PGS of Oncopeltus fasciatus- The anterior-right head region of germband stage embryos, stained for α-tubulin (green) and DAPI (blue). Ages in hours after egg laying (hEAL) progress from top to bottom. In each row, the left panel is a full projection of a confocal stack (column 1), and columns 2-5 are increasingly deep optical slices. Viewed ventrally, anterior towards the top. Morphological structures outlined by dashed lines, morphologically distinct neuroblasts (>8µm in diameter) marked by asterisks, cells with visibly dividing nucleus (DAPI) marked by arrowheads.

**Supplementary Figure 3B:** Series of α-tubulin antibody-stained digital sections of the PGS of Oncopeltus fasciatus- α-tubulin channel. The anterior-right head region of germband stage embryos. Ages in hours after egg laying (hEAL) progress from top to bottom. In each row, the left panel is a full projection of a confocal stack (column 1), and columns 2-5 are increasingly deep optical slices. Viewed ventrally, anterior towards the top. Morphological structures outlined by dashed lines, morphologically distinct neuroblasts (>8µm in diameter) marked by asterisks.

**Supplementary Figure 3C:** Series of DAPI antibody-stained digital sections of the PGS of Oncopeltus fasciatus- DAPI channel. The anterior-right head region of germband stage embryos. Ages in hours after egg laying (hEAL) progress from top to bottom.In each row, the left panel is a full projection of a confocal stack (column 1), and columns 2-5 are increasingly deep optical slices. Viewed ventrally, anterior towards the top. Morphological structures outlined by dashed lines, cells with visibly dividing nucleus (DAPI) marked by arrowheads.

**Supplementary Figure 4A:** Series of Ph3 antibody-stained digital sections of the PGS of Oncopeltus fasciatus – Ph3 channel. The anterior head region of germband stage embryos. Columns 1-3 show increasingly deeper optical sections. Anterior towards the top.

**Supplementary Figure 4B:** Series of Ph3 antibody-stained digital sections of the PGS of Oncopeltus fasciatus - DAPI channel. The anterior head region of germband stage embryos. Columns 1-3 show increasingly deeper optical sections. Anterior towards the top.

**Supplementary Figure 4C:** A graph quantifying the number of PH3+ cells per 100μm² square in the brain regions compared to the rest of the head in the slices shown in figure 4. Segmenting and PH3+ positive cell counting were done manually for each slice, then summed up per age (3 slices per age) and divided by total segment area (brain or rest of the sample).

**Supplementary Figure 5A:** A timed series of mRNA In-Situ hybridization stain during mid to late germband formation stage in Oncopeltus, focusing on the PGS. The columns show embryos of the same age, and the rows show embryos stained for the same gene. Anterior towards the top. The head regions are shown in each series, in the same order, although magnified regions are not always from the same specimen as in main figure 5, as they have been selected for highest quality of magnification. *Delta* expression marks cells with neurogenic potential and neuroblasts. *Snail* marks asymmetrically dividing and migrating neuroblasts. *ash* marks proliferating neuroblasts, *pros* marks differentiating neuroblasts, and *elav* marks neurons. Scale bar 100μm.
